# Effects of ezetimibe/simvastatin 10/10 mg versus Rosuvastatin 10 mg on carotid atherosclerotic plaque inflammation

**DOI:** 10.1186/s12872-019-1184-2

**Published:** 2019-08-19

**Authors:** Minyoung Oh, Hyunji Kim, Eon Woo Shin, Changhwan Sung, Do-Hoon Kim, Dae Hyuk Moon, Cheol Whan Lee

**Affiliations:** 1 Department of Nuclear Medicine, Asan Medical Center, University of Ulsan College of Medicine, Seoul, South Korea; 2 Division of Cardiology, Heart Institute, Asan Medical Center, University of Ulsan College of Medicine, Seoul, South Korea

**Keywords:** Ezetimibe, Plaque inflammation, Statin, Positron emission tomography

## Abstract

**Background:**

Using ^18^F-fluorodeoxyglucose (^18^FDG) positron emission tomography-computed tomography (PET/CT) imaging, we examined the effects of ezetimibe/simvastatin 10/10 mg versus rosuvastatin 10 mg on carotid atherosclerotic plaque inflammation. Whether the combination therapy of ezetimibe with low-dose statin is as effective as potent statin monotherapy in attenuating carotid atherosclerotic plaque inflammation remains unclear.

**Methods:**

In this 2-by-2 factorial trial, 50 patients with ^18^FDG uptake (target-to-background ratio [TBR] ≥1.6) in the carotid artery and acute coronary syndrome were randomized to receive either simvastatin/ezetimibe 10/10 mg or rosuvastatin 10 mg. ^18^FDG PET/CT examinations were performed at baseline and at 6 months. The percent change in the TBR of the index vessel at the most diseased segment (MDS) was the primary endpoint.

**Results:**

Baseline characteristics of the two groups were largely similar. At 6-month follow-up, the MDS TBR of the index vessel and aorta significantly decreased in ezetimibe/simvastatin group and tended to decrease in rosuvastatin group. However, the percent change in the MDS TBR of the index vessel was similar between the 2 groups (− 10.22 ± 17.49% vs. -5.84 ± 15.78%, respectively, *p* = 0.357), as was the percent change in the whole vessel TBR of the index vessel. Likewise, the changes in the MDS TBR or whole vessel TBR of the aorta were similar in both groups. Total cholesterol and low-density lipoprotein cholesterol levels improved to a similar degree in both groups.

**Conclusion:**

Treatment with ezetimibe/simvastatin versus rosuvastatin resulted in a similar improvement of carotid atherosclerotic plaque inflammation, suggesting their equivalent anti-inflammatory effects.

**Trial registration:**

The trial is registered at ClinicalTrials.gov: NCT02378064, 3-4-2015. /IRB No. 2015–0194.

## Background

Statins have been extensively studied in both primary and secondary prevention trials, and statin therapy has been shown to reduce the risk of death and cardiovascular events in a broad range of patient populations [1–3]. There is a linear relationship between the magnitude of low-density lipoprotein (LDL)-cholesterol reduction and the magnitude of cardiovascular risk reduction, indicating that statins exert their beneficial effects primarily by decreasing LDL cholesterol [1, 2, 4]. In addition, the overall benefits of statin therapy seem to exceed that which might be expected from changes in LDL-cholesterol levels alone [5–8]. Statins not only inhibit cholesterol biosynthesis but also the biosynthesis of isoprenoids, which might be implicated in endothelial dysfunction and vascular inflammation [7]. Furthermore, statins lower C-reactive protein levels, which suggests that the efficacy of statins might be partly due to their anti-inflammatory effects [3, 9–11]. In recent years, however, large-scale randomized controlled trials with non-statin cholesterol-lowering therapies have shown similar benefits to statins in reducing the risk of cardiovascular events [12, 13], thereby raising questions about potentially unique pleiotropic properties of statins. Indeed, it is unclear whether statins have effects other than those that lower LDL cholesterol that may suppress atherosclerotic plaque inflammation.

Statin side effects are related to the dose or potency of the given drugs [14, 15], and a combination therapy of ezetimibe with low-dose statin is occasionally used to minimize adverse effects. However, there is little information about whether this approach is as effective as potent statin monotherapy in decreasing LDL cholesterol levels and attenuating atherosclerotic plaque inflammation. Using ^18^F-fluorodeoxyglucose (^18^FDG) positron emission tomography (PET) imaging, we examined the effects of ezetimibe/simvastatin 10/10 mg versus rosuvastatin 10 mg on carotid atherosclerotic plaque inflammation in patients with acute coronary syndrome.

## Methods

Between May 2015 and December 2017, we conducted a single center, randomized, open label trial using a 2-by-2 factorial design (ClinicalTrials.gov number, NCT02378064). The trial evaluated cholesterol-lowering therapy with ezetimibe/simvastatin 10/10 mg versus rosuvastatin 10 mg and blood pressure-lowering therapy with fimasartan versus amlodipine in patients with acute coronary syndrome. The results of the blood pressure-lowering therapy have been previously reported in another study, in which detailed information as to the inclusion and exclusion criteria were described [16]. In brief, patients were eligible if they had history of hypertension (or blood pressure ≥ 140/90 mmHg at baseline), acute coronary syndrome, and at least one ^18^FDG uptake lesion in the carotid artery (target-to-background ratio [TBR] ≥1.6) according to ^18^FDG PET/CT imaging. Exclusion criteria included patients 1) scheduled for carotid endarterectomy or stenting, 2) with chronic disease that needed to be treated with oral, intravenous, or intraarticular steroid, 3) who had used RAS or calcium channel blocker therapy in the past 4 weeks, 4) with congestive heart failure or left ventricular ejection fraction less than 40%, 5) with chronic renal failure (serum creatinine > 2.0 mg/dl or creatinine < 40 ml/min (by Cockcroft-Gault method), 6) with chronic liver disease, and 7) with type I diabetes.

Baseline ^18^FDG PET/CT examination was done within percutaneous coronary intervention (3–5 days after admision) or 2 days of coronary angiography. Eligible patients were randomly assigned to the ezetimibe/simvastatin group (10/10 mg once a day for 6 months) or the rosuvastatin group (10 mg once a day for 6 months) groups using computer-generated random numbers. All patients were treated with standard medications including blood pressure-lowering therapy and antiplatelet agents. Six-month follow-up ^18^FDG PET/CT examination was performed in all patients. Biochemical laboratory tests were done at admission and at 6-month follow-up. Our Institutional Review Committee approved the study protocol (No. 2015–0194). All patients provided written informed consent prior to enrollment in accordance with the 1975 Declaration of Helsinki.

Before being scanned, the patients fasted for at least 8 h. Blood glucose levels were maintained below 130 mg/dL. Patients with diabetes mellitus adhered to their glucose-lowering medication regularly as prescribed. All patients were examined ^18^FDG PET/CT using Discovery 690 PET/CT scanner (GE, Waukesha, WI, USA) with time-of-flight capability in accordance with previous reports [17, 18]. Two hours after the ^18^FDG injection (5.2 MBq [0.14 mCi]/kg body weight), a three-dimensional PET/CT scan was started. CT was performed first to correct scattering and photon attenuation using a continuous spiral 64-slice technique with a voltage of 140 kV, a current of 200 mA, a pitch of 0.98 (39.4 mm/rotation), a rotation speed of 0.4 s/revolution and a slice thickness of 2.5 mm. PET was performed immediately afterwards with an axial field of view of 15.7 cm. And images were acquired from the cranial base to the upper thorax obtained for 10 min/bed. Images were reconstructed with the three-dimensional ordered-subsets expectation maximization reconstruction algorithm (4 iterations, 18 subsets) with matrix of 256 × 256 after CT-based scattering correction and attenuation correction.

A dedicated workstation was used for analysis of images. PET images were evaluated whether focal ^18^FDG activity in the ascending aorta and bilateral carotid arteries is present by visual inspection. On every slice of the axial PET/CT images, arterial ^18^FDG activity was determined by creating a circular region-of-interest (ROI) containing the arterial wall and the lumen. The maximal standardized uptake values (SUVs) of each ROI were measured as the maximal pixel activity for each slice adjusted for injected ^18^FDG dose and the lean body mass. The maximal SUVs for each artery were calculated by averaging the SUVs of all slices within an arterial territory. The SUVs were normalized to venous ^18^FDG activity by dividing them by the average venous ROI estimated from the superior vena cava, which yielded an arterial target to background ratio (TBR).

The most diseased segment (MDS) TBR was assessed by centering on the slice of the artery with the maximal ^18^FDG activity and then averaging contiguous 5 segments. The whole vessel TBR was assessed as the mean of the maximal TBR activity for all segments of each vessel. To describe ^18^FDG-defined atherosclerotic inflammation activity, whole vessel ^18^FDG activity (TBR) was assessed in the 3 target arteries (bilateral carotid arteries and aorta) and used. Due to diverse impact of catheter-related aortic injury during cardiac catheterization, and the one of the carotid arteries with the highest ^18^FDG activity was chosen as the index vessel at baseline [18].

The percent change in the MDS TBR of the index vessel calculated as (MDS TBR at 6 months – MDS TBR at baseline) / (MDS TBR at baseline) × 100 was defined as the primary endpoint. Secondary endpoints were changes in lipid profiles [total cholesterol, triglyceride, high-density lipoprotein (HDL) cholesterol, and LDL cholesterol], systolic/diastolic blood pressure, and high-sensitivity C-reactive protein.

A sample of 22 participants per treatment group was estimated to provide the 90% power to detect a 15% difference in the primary endpoint between the rosuvastatin and ezetimibe/simvastatin groups (assuming a SD of 15% in each group) with a significance level of 0.05, using a two-sided test. With an anticipated dropout rate of 10%, total 50 patients (25 patients in each group) was necessary to provide an adequate number of evaluable patients. Categorical variables were expressed as frequencies, whereas continuous variables as means ± standard deviations or medians with interquartile ranges. The paired *t*-test or Wilcoxon rank sum test were used to compare the changes of continuous variables in each group, and the unpaired *t*-test or Mann-Whitney U-test for differences between groups. An analysis with two-sided *p*-value < 0.05 was considered statistically significant.

## Results

**Fig. 1:**
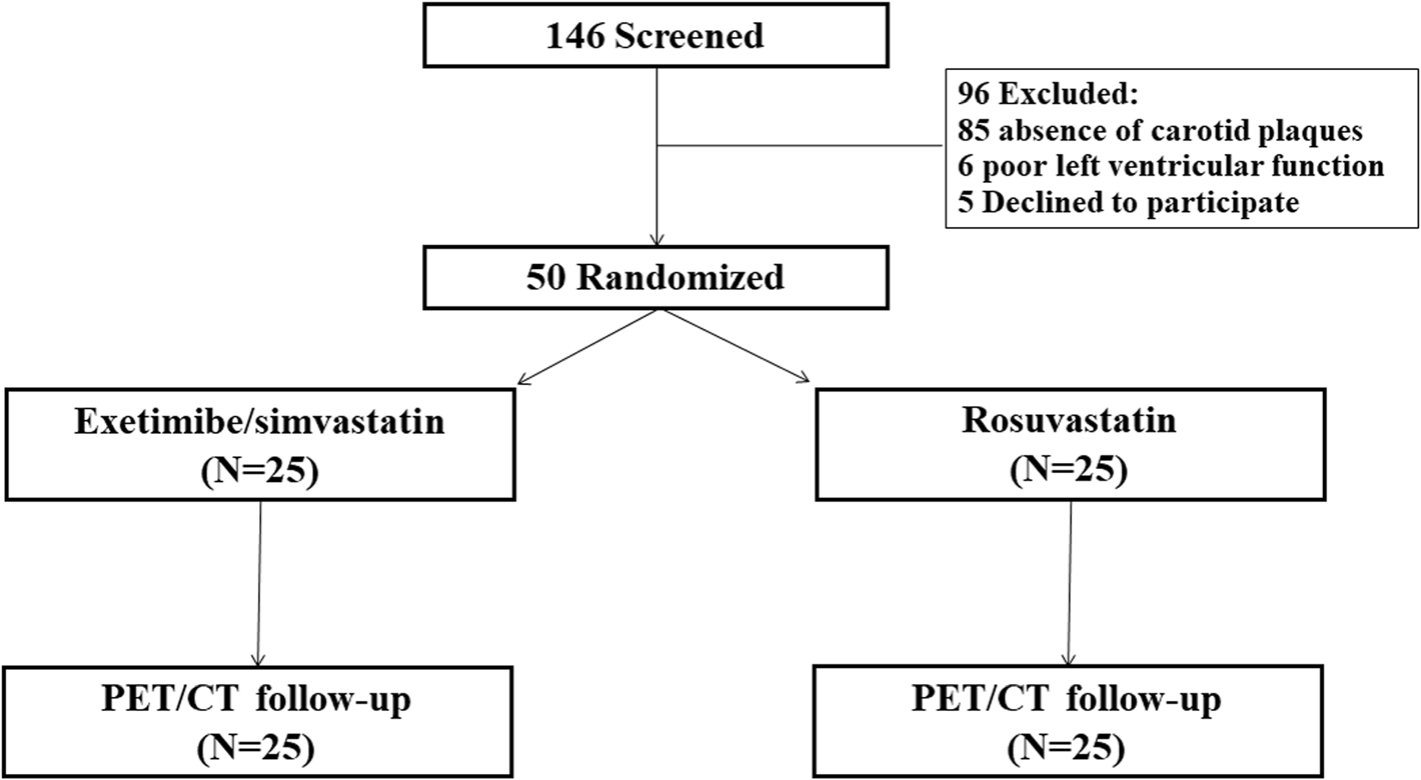
Study flowchart of patient enrollment

Among the 146 screened patients with acute coronary syndrome, 96 did not fulfill the eligibility criteria for the present study, and 50 patients were eventually randomized to either the ezetimibe/simvastatin group or the rosuvastatin group. Exclusion was due to poor left ventricular function (*n* = 6), the absence of carotid atherosclerosis (*n* = 85), and patient refusal (*n* = 5) (Fig. 1). Six-month follow-up PET/CT examination was performed in all patients.

The baseline characteristics were largely similar between the two groups (Table 1). The mean age of the patients was 60.9 ± 8.2 years, the mean systolic blood pressure was 145.5 ± 14.21 mmHg, and the mean LDL cholesterol level was 118.9 ± 34.52 mg/dL (Table 2). Men comprised 86% of the patients. Clinical presentations were non-ST-segment elevation acute coronary syndrome in 24.0% of the patients, and ST-segment elevation myocardial infarction in 76.0% of the patients and. Percutaneous coronary intervention was performed in most patients (98.0%), except one patient (2.0%) with medications.

**Table 1:** Baseline Clinical characteristics

Characteristics	Ezetimibe /simvastatin (*n* = 25)	Rosuvastatin (*n* = 25)	*p*-value
Age, years	62.5 ± 7.4	59.2 ± 8.8	0.154
Men	22 (88.0%)	21 (84.0%)	0.684
Current smoker	4 (16.0%)	5 (20.0%)	0.713
Diabetes mellitus	2 (8.0%)	3 (12.0%)	0.637
Hypertension	17 (68.0%)	10 (40.0%)	0.047
Diagnosis			0.301
STEMI	19 (76.0%)	19 (76.0%)	
NSTE-ACS	6 (2.0%)	6 (24.0%)	
Culprit artery of ACS			0.345
Left anterior descending coronary	17 (68.0%)	16 (64.0%)	
Left circumflex coronary	2 (8.0%)	0 (0%)	
Right coronary	6 (24.0%)	8 (32.0%)	
Ramus intermedius	0 (0%)	1 (4.0%)	
Culprit lesion PCI	24 (97.5%)	25 (100%)	0.848
Left ventricular ejection fraction (%)	52.9 ± 8.2	53.9 ± 73	0.718
Medications at the time of follow-up			
Aspirin	25 (100%)	25 (100%)	1.0
P2Y12 inhibitors	25 (100.0%)	25 (100.0%)	1.0
β-blockers	22 (88.0%)	19 (76.0%)	0.269
Angiotensin II receptor blocker	12 (48.0%)	13 (52.0%)	0.777
Calcium channel blocker	13 (52.0%)	12 (48.0%)	0.777

*CAD* Coronary artery disease, *STEMI* ST-Segment elevation myocardial infarction, *NSTE-ACS* Non-ST-segment elevation-acute coronary syndrome, *PCI* Percutaneous coronary intervention

**Table 2:** Laboratory Findings

Characteristics	Ezetimibe /simvastatin (n = 25)	Rosuvastatin (n = 25)	*p*-value
Total cholesterol (mg/dl)			
Baseline	174.2 ± 38.90	178.2 ± 31.80	0.689
6 months	135.6 ± 23.65	129.4 ± 23.37	0.359
Triglyceride (mg/dl)			
Baseline	110.7 ± 46.88	115.0 ± 56.11	0.771
6 months	113.8 ± 43.70	115.2 ± 44.93	0.914
LDL cholesterol (mg/dl)			
Baseline	114.3 ± 34.83	123.5 ± 34.29	0.349
6 months	87.3 ± 20.00	81.1 ± 21.9	0.301
HDL cholesterol (mg/dl)			
Baseline	45.5 ± 8.93	45.7 ± 10.06	0.941
6 months	45.2 ± 7.95	46.7 ± 8.32	0.490
Hs-CRP (mg/L)			
Baseline	0.34 ± 0.68	0.38 ± 0.43	0.801
6 months	0.10 ± 0.17	0.11 ± 0.24	0.886

*Hs-CRP* High sensitivity C-reactive protein

Lipid profiles, blood pressure, and high-sensitivity C-reactive protein levels at baseline were similar between the 2 groups (Table 2). Total cholesterol and LDL cholesterol levels significantly decreased in both groups at 6-month follow-up (*p* < 0.001). High sensitivity C-reactive protein levels significantly decreased in the rosuvastatin group (*p* = 0.016) and tended to decrease in the ezetimibe/simvastatin group (*p* = 0.090). However, HDL cholesterol and triglyceride levels did not significantly change in either group. Likewise, blood pressure changes were not different between the 2 groups (systolic: 17.7 ± 13.38% for the rosuvastatin group vs. 15.8 ± 15.72% for the ezetimibe/simvastatin group; *p* = 0.650; diastolic: 15.8 ± 17.18% vs. 12.3 ± 17.39%, respectively; *p* = 0.481).

**Fig. 2:**
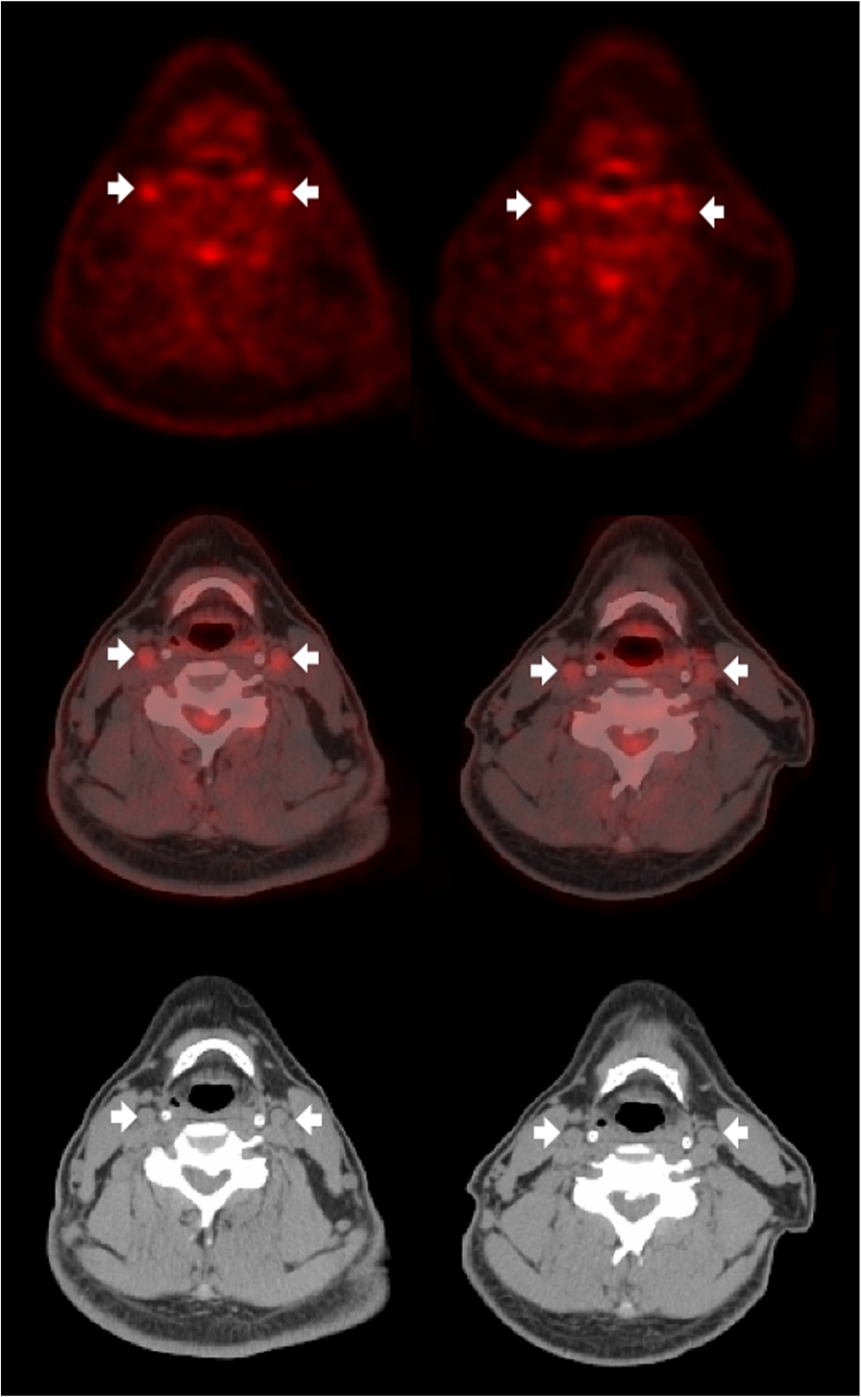
^18^FDG uptakes of the index vessel in a patient treated with ezetimibe/simvastatin (arrows). Representative CT (top), ^18^FDG-PET (middle), and ^18^FDG-PET/CT (bottom) images at baseline (left) and at the 6-month follow-up (right) are shown. ^18^FDG uptakes markedly decreased at the 6-month follow-up

**Fig. 3:**
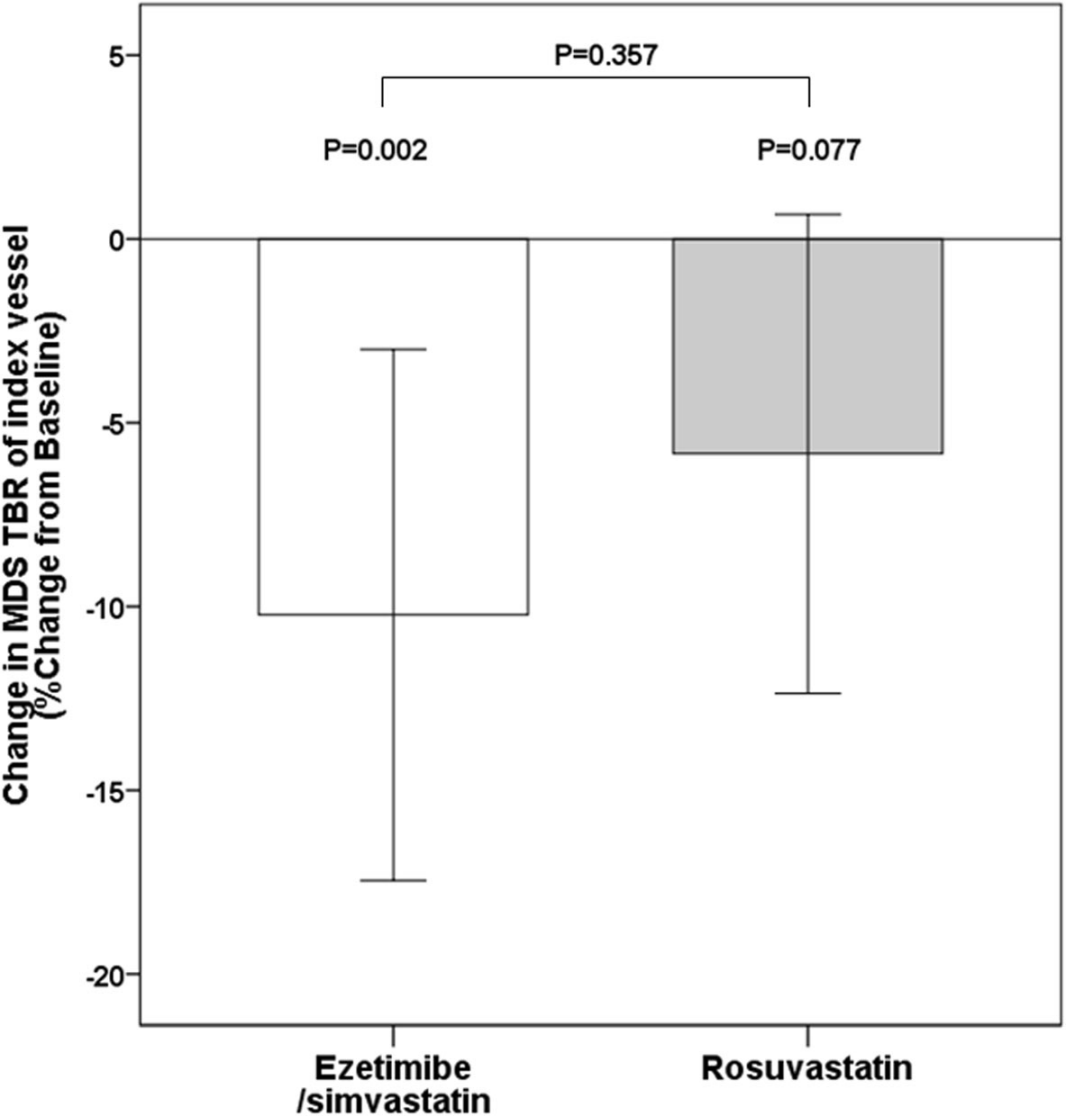
Change in MDS (most diseased segment) TBR (target-to-background ratio) of the index vessel. The percent change in MDS TBR of the index vessel at 6-month follow-up was similar between the 2 groups

Figure. 2 shows representative images of improved ^18^FDG uptake in the carotid plaque after ezetimibe/simvastatin therapy. As summarized in Table 3, baseline ^18^FDG PET/CT parameters were similar between the 2 groups. The MDS TBR of the index vessel at 6-month follow-up significantly decreased in the ezetimibe/simvastatin groups (*p* = 0.002) and tended to decrease in the rosuvastatin group (*p* = 0.077). However, the percent change in the MDS TBR of the index vessel (primary endpoint) was not significantly different between both groups (− 10.22 ± 17.49% vs. -5.84 ± 15.78%, respectively, *p* = 0.357) (Fig. 3). Similarly, the MDS TBR of the ascending aorta significantly decreased in the ezetimibe/simvastatin groups (p = 0.002) and tended to decrease in the rosuvastatin group (*p* = 0.052). The percent change in the whole vessel TBR of the index vessel did not differ between the 2 groups. Similar results were detected for changes in the MDS TBR and whole vessel TBR of the aorta. No significant correlations were found between changes in the lipid profile, C-reactive protein levels, or blood pressure and percent changes in the MDS TBR of the index vessel.

**Table 3:** Changes in Arterial Inflammation Activity: Index Vessel Analysis

Characteristics	Ezetimibe /simvastatin (n = 25)	Rosuvastatin (n = 25)	*p-*value between groups
MDS TBR of index carotid artery			
Baseline	2.37 ± 0.46	2.23 ± 0.41	0.271
Follow-up	2.07 ± 0.29	2.09 ± 0.46	0.889
Nominal change	−0.30 ± 0.43	−0.15 ± 0.40	0.197
*p*-value compared with baseline	0.002	0.077	
Percent change (primary endpoint)	−10.22 ± 17.49	−5.84 ± 15.78	0.357
Whole vessel TBR of index carotid artery			
Baseline	2.00 ± 0.0.39	1.94 ± 0.31	0.537
Follow-up	1.81 ± 0.26	1.83 ± 0.35	0.772
Nominal change	−0.19 ± 0.38	− 0.11 ± 0.38	0.419
*p*-value compared with baseline	0.017	0.168	
Percent change	−6.81 ± 19.06	−3.95 ± 17.01	0.579
MDS TBR of aorta			
Baseline	2.58 ± 0.45	2.57 ± 0.45	0.912
Follow-up	2.27 ± 0.34	2.35 ± 0.46	0.459
Nominal change	−0.31 ± 0.44	−0.21 ± 0.52	0.466
*p*-value compared with baseline	0.002	0.052	
Percent change	−10.35 ± 16.24	−6.80 ± 18.36	0.473
Whole vessel TBR of aorta			
Baseline	2.47 ± 0.43	2.47 ± 0.45	0.959
Follow-up	2.19 ± 0.33	2.26 ± 0.45	0.507
Nominal change	−0.29 ± 0.43	−0.20 ± 0.52	0.549
*p*-value compared with baseline	0.003	0.063	
Percent change	−9.580 ± 16.45	−6.58 ± 19.04	0.526

Nominal change is calculated as follow-up minus baseline, and percent change as (follow-up minus baseline)/baseline×100. *MDS* Most diseased segment, *TBR* Tissue blood ratio

## DISCUSSION

In this study, we found that in patients with carotid artery disease and acute coronary syndrome, both ezetimibe/simvastatin 10/10 mg and rosuvastatin 10 mg improved carotid atherosclerotic plaque inflammation without between-group differences. Aortic inflammation was also similarly decreased in both groups. Likewise, changes in the serum levels of total cholesterol, LDL cholesterol, and high-sensitivity C-reactive protein were not different in both groups. These findings suggest that treatment with ezetimibe plus low-dose statin versus potent statin monotherapy offers comparable anti-inflammatory effects when administered at equivalent daily doses.

Statins remains the medicine of choice for cardiovascular risk reduction. For patients with clinical atherosclerotic cardiovascular disease or diabetes mellitus, moderate- or high-intensity statin therapy is primarily recommended [19]. In real-word practice, however, an ezetimibe plus low-intensity statin regimen is occasionally prescribed to treat these patients owing to concerns about the side effects of statins. The benefits observed with statin therapy may not be attributed entirely to their cholesterol-lowering properties but also to pleiotropic effects. However, it is unclear whether the combination therapy of ezetimibe with low-intensity statin has similar pleiotropic effects compared with potent statin monotherapy to yield the same degree of LDL cholesterol reduction. Previously, simvastatin/ezetimibe 10/10 mg and rosuvastatin 10 mg at equivalent LDL cholesterol-lowering doses were shown to similarly reduce plasma markers of oxidative stress and inflammation activity [20]. In the present study, there was no difference between the 2 regimens in reducing carotid atherosclerotic plaque inflammation, suggesting equivalent anti**-**inflammatory effects. These findings support the current clinical practice of reducing LDL cholesterol using a combination of ezetimibe plus low-intensity statin.

Ezetimibe selectively blocks intestinal absorption of dietary and biliary cholesterol and promotes a compensatory increase in cholesterol synthesis [21]. As a result, ezetimibe leads to a substantial additional reduction in LDL cholesterol levels when added to statin therapy [22]. However, the question of whether ezetimibe shares similar anti-atherosclerotic properties with statins has been debated [23]. In the Ezetimibe and Simvastatin in Hypercholesterolemia Enhances Atherosclerosis Regression (ENHANCE) trial [24], combination therapy with ezetimibe/simvastatin did not show a significant difference in intima-media thickness versus the use of simvastatin alone. In contrast, ezetimibe/fluvastatin combination therapy was found to increase the fibrous cap thickness of lipid-rich plaque, as compared to fluvastatin monotherapy [25]. In the PRECISE-IVUS study, ezetimibe/atorvastatin resulted in a more remarkable reduction of LDL cholesterol compared to atorvastatin monotherapy, with favorable effects on coronary atherosclerotic plaque [26]. Furthermore, the combination of ezetimibe and simvastatin versus simvastatin monotherapy resulted in the incremental lowering of LDL cholesterol levels and improved cardiovascular outcomes [12]. Overall, an ezetimibe plus low-intensity statin or potent statin alone at equivalent LDL cholesterol-lowering doses appears to have comparable anti-atherosclerotic effects. These findings are also compatible with previous studies showing that the clinical benefit of cholesterol lowering therapies mostly depends on the absolute reduction in LDL cholesterol and the total duration of therapy [27, 28].

Several potential limitations of the study need to be addressed. First, the number of study subjects was relatively small, which may not have allowed for sufficient power to detect a subtle difference in the MDS TBR of the index vessel. Second, an open-label design is subject to inherent limitations. We tried to overcome the limitations by using blind ^18^FDG PET/CT evaluations. Third, a placebo arm was not included owing to ethical considerations. Finally, the results of the paper are not generalizable to all patients with acute coronary syndrome or at high risk for cardiovascular events, but to those who cannot tolerate at least moderate-intensity statin therapy.

## Conclusion

In this study, we found that both ezetimibe/simvastatin 10/10 mg and rosuvastatin 10 mg resulted in a similar improvement of carotid atherosclerotic plaque inflammation in patients with carotid artery disease and acute coronary syndrome. It suggests that their anti-inflammatory effects are equivalent.

## Data Availability

The datasets generated and analysed during the current study are not publicly available due to patient confidentiality but are available from the corresponding author on reasonable request.

## Abbreviations

^18^FDG: ^18^F-fluorodeoxyglucose
CT: Computed tomography
HDL: High density lipoprotein
LDL: Low-density lipoprotein
MDS: Most diseased segment
PET: Positron emission tomography
ROI: Region-of-interest
SUVs: Standardized uptake values
TBR: Target to background ratio
